# Effective Depletion of Pre-existing Anti-AAV Antibodies Requires Broad Immune Targeting

**DOI:** 10.1016/j.omtm.2017.01.003

**Published:** 2017-01-25

**Authors:** Victoria M. Velazquez, Aaron S. Meadows, Ricardo J. Pineda, Marybeth Camboni, Douglas M. McCarty, Haiyan Fu

**Affiliations:** 1 Center for Vaccine and Immunology, Research Institute at Nationwide Children’s Hospital, Columbus, OH 43205, USA; 2 Center for Gene Therapy, Research Institute at Nationwide Children’s Hospital, Columbus, OH 43205, USA; 3 Department of Pediatrics, School of Medicine, The Ohio State University, Columbus, OH 43210, USA

**Keywords:** AAV, pre-existing antibody, gene therapy

## Abstract

Pre-existing antibodies (Abs) to AAV pose a critical challenge for the translation of gene therapies. No effective approach is available to overcome pre-existing Abs. Given the complexity of Ab production, overcoming pre-existing Abs will require broad immune targeting. We generated a mouse model of pre-existing AAV9 Abs to test multiple immunosuppressants, including bortezomib, rapamycin, and prednisolone, individually or in combination. We identified an effective approach combining rapamycin and prednisolone, reducing serum AAV9 Abs by 70%–80% at 4 weeks and 85%–93% at 8 weeks of treatment. The rapamycin plus prednisolone treatment resulted in significant decreases in the frequency of B cells, plasma cells, and IgG-secreting and AAV9-specific Ab-producing plasma cells in bone marrow. The rapamycin plus prednisolone treatment also significantly reduced frequencies of IgD^−^IgG^+^ class-switched/FAS^+^CL7^+^ germinal center B cells, and of activated CD4^+^ T cells expressing PD1 and GL7, in spleen. These data suggest that rapamycin plus prednisolone has selective inhibitory effects on both T helper type 2 support of B cell activation in spleen and on bone marrow plasma cell survival, leading to effective AAV9 Abs depletion. This promising immunomodulation approach is highly translatable, and it poses minimal risk in the context of therapeutic benefits promised by gene therapy for severe monogenetic diseases, with a single or possibly a few treatments over a lifetime.

## Introduction

Adeno-associated virus (AAV) vectors are promising as effective gene delivery tools for long-term transduction in a broad range of tissues. They have displayed efficacy and safety after systemic delivery in numerous pre-clinical disease models and in clinical trials, particularly for monogenic diseases.^1^, ^2^, ^3^, ^4^, ^5^, ^6^ The recognition of the trans-blood-brain barrier neurotropic properties of AAV9 vector^7^, ^8^ have led to significant advancements in AAV gene delivery for diseases with global or broad neuropathies in the CNS, demonstrating promising clinical potential.^4^, ^5^, ^6^ These studies have led to the translation of systemic AAV9 gene delivery to a phase 1 clinical trial in patients with type 1 spinal muscular atrophy (SMA1) (NCT02122952) and phase 1/2 trials in patients with MPS IIIA (NCT02716246, ongoing) and MPS IIIB (to be initiated) and intrathecal gene delivery clinical trials in patients with giant axonal neuropathy (NCT02362438) and Batten disease (CLN6) (NCT02725580).

As effective AAV gene therapy approaches become available for clinical application, pre-existing host humoral immunity against AAV poses critical challenges. While having no known pathogenesis, AAV is widespread in humans and more than 90% of the adult population is naturally infected, with a high prevalence of antibodies (Abs) to various AAV serotypes.^9^, ^10^ Although AAV2 is the most prevalent serotype in humans, cross-reactivity among different serotypes^9^, ^10^, ^11^ reduces the potential utility of AAV vectors packaged in alternative serotypes. In addition, potent anti-AAV immunity arises following recombinant AAV (rAAV) gene therapy treatments, making re-administration currently unfeasible. Notably, while the presence of neutralizing Abs (nAbs) against specific AAV serotypes that is generally used is a critical exclusion criteria in clinical trials, non-neutralizing anti-AAV Abs also can trigger vector clearance.^12^

No effective approaches are currently available to overcome pre-existing AAV Abs. Various clinically relevant models have been established to characterize pre-existing anti-AAV humoral immunity and cross-reactivity.^11^, ^12^, ^13^, ^14^, ^15^, ^16^ Efforts have been made to develop strategies to overcome pre-existing anti-AAV Abs, including AAV capsid modification and decoys,^17^, ^18^, ^19^, ^20^ transient pharmacological immunomodulation,^21^, ^22^, ^23^, ^24^, ^25^ and plasmapheresis,^24^, ^26^ demonstrating potential for improving rAAV gene delivery by circumventing or reducing the pre-existing Abs in animal models and in humans. While capsid modification strategies are under development, pharmacological immunomodulation agents are readily available for testing. Short-term transient immunosuppression (IS) regimens were tested in previous studies, using chemical IS agents and specific Abs individually or in combinations, including cyclosporine A, mycophenolate mofetil, tacrolimus, Rituximab, sirolimus, methylprednisolone, CTLA4Ig, non-depleting CD4 Ab, and T cell-depleting anti-thymocyte gamma-globulin (ATG), all of which target specific immune cells or signals. The majority of these studies showed some potential for IS regimens in improving rAAV transduction,^21^, ^22^, ^23^ while others had no impact on pre-existing AAV Abs.^24^, ^25^, ^27^

While the mechanisms are not clear, it is well established that very complex anatomical niches, molecular signals, and cellular components are required for the priming and maintenance of long-lived Ab-producing plasma cells (PCs).^28^, ^29^, ^30^, ^31^ We therefore believe that effectively diminishing pre-existing Abs requires broad immune system targeting. In this study, we tested multiple IS agents individually or in combination in a mouse model with pre-existing anti-AAV9 Abs, and we identified an effective regimen for depleting pre-existing Abs through a combination of the mTOR inhibitor rapamycin (Rap) and the classical broad IS agent prednisolone (Pred).

## Results

To identify effective approaches for depleting pre-existing Abs, we generated a mouse model of pre-existing Abs against AAV9 by immunizing 6- to 8-week-old wild-type (WT) mice with an intraperitoneal (i.p.) injection of rAAV9-CMV-hNAGLU vector. At 4 weeks post-immunization (pi), serum samples were assayed by ELISA for total anti-AAV9 IgG. Beginning at 5 weeks pi, we began treatment of AAV9-immunized mice with approved IS drugs Rap, bortezomib (Bort), and Pred, individually or in combination at different doses. Blood samples were assayed by binding ELISA for anti-AAV9 IgG after 4 and 8 weeks on IS treatments. Necropsies were performed after 8 weeks on IS, and spleen and bone marrow samples were collected for analyses. Non-IS-treated AAV9-immunized mice (n = 5) and naive (n = 2) WT mice were used as controls. Table 1 summarizes the study design.

### Effective Depletion of Pre-existing Anti-AAV9 Abs by Broad Immune Targeting Using a Combination of Rapamycin and Prednisolone

**Figure 1:**
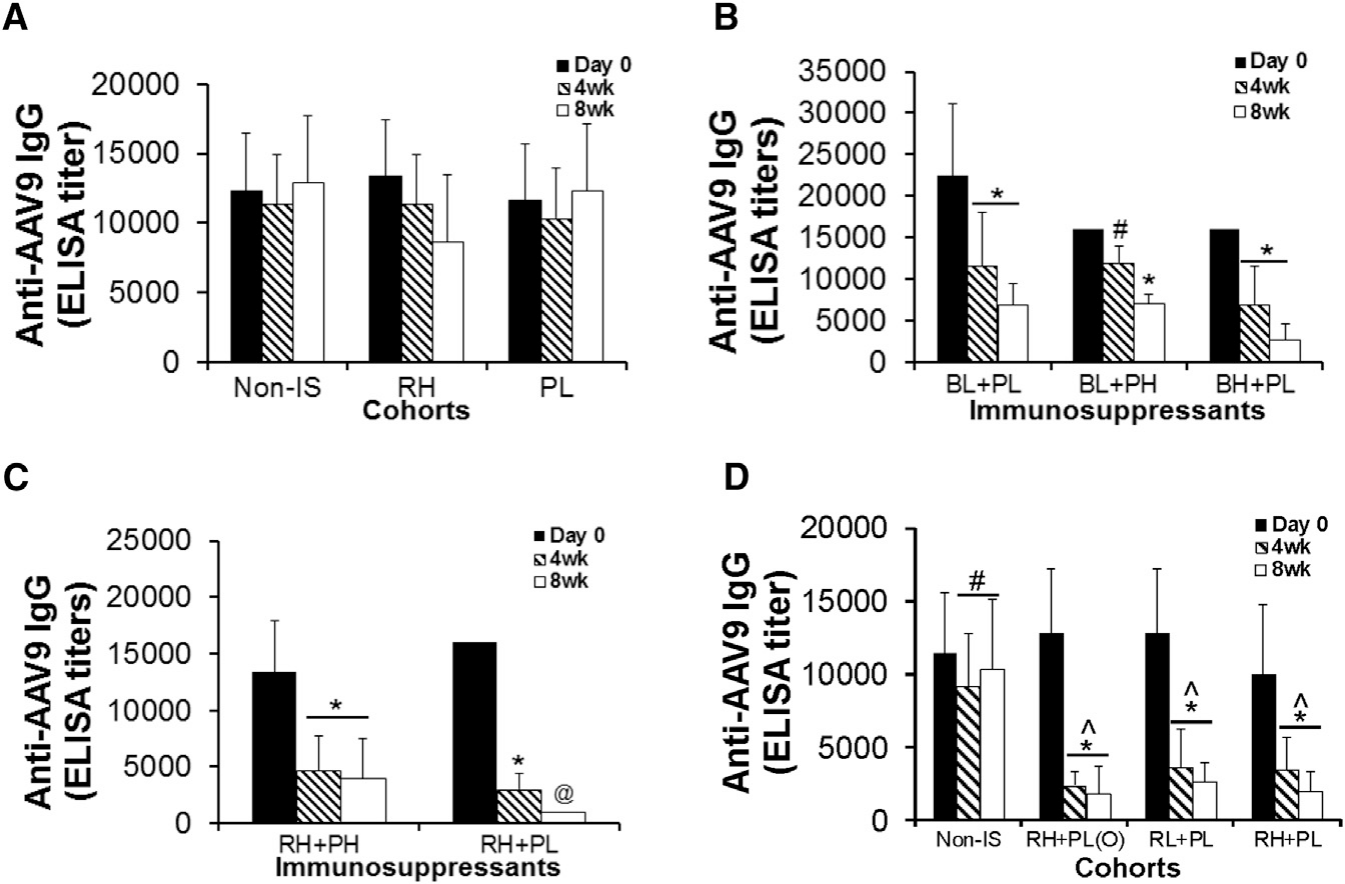
Significant Reduction of Pre-existing Antibodies against AAV9 in Mice by Immunosuppression Regimens (A–D) WT mice were immunized with an i.p. injection of rAAV9 vector, and then they were treated with selected immunosuppressants (IS), beginning at 4 weeks pi. Serum samples (n = 3–13/group) from IS-treated animals and non-IS controls were assayed for anti-AAV9-IgG at day 0, 4 weeks, and 8 weeks of IS treatment. (A) Treatment with Rap or Pred only is shown. (B) Dose response of Bort + Pred treatment is shown. (C) Dose response of Rap + Pred treatment is shown. (D) Rap + Pred: dose response and administration route of pred are shown. Pre-IS, prior to IS treatment; non-IS, non-IS controls; RH, high dose of rapamicin, i.p.; RL, low dose of rapamicin, i.p.; BH, high dose of bortezomib, i.p.; BL, low dose of bortezomib, i.p.; PH, high dose of prednisolone, i.p.; PL, low dose of prednisolone; PL(O), oral low dose of prednisolone. *p ≤ 0.05 versus pre-IS treatment; #p ≥ 0.05 versus pre-IS treatment; ˆp ≤ 0.05 versus non-IS; @n = 2.

To identify an effective IS regimen for the depletion of pre-existing anti-AAV Abs, we treated AAV9-immunized mice with Rap, Bort, and Pred, all by i.p. injection, individually or in combination at different doses (Table 1, Experiment 1). The data showed no significant differences in serum anti-AAV9-IgG levels in mice that received Rap (Figure 1A), Pred (Figure 1A), or Bort (data not shown) individually, after 8 weeks on treatments. However, using a combination of Rap or Bort with Pred resulted in a significant reduction in serum AAV9 Ab levels (Figures 1B–1D). Notably, Rap + Pred treatments were more effective in reducing serum anti-AAV9 Abs, by 70%–80% at 4 weeks and 85%–93% at 8 weeks on the treatment (Figure 1C), compared to a 25%–50% decrease at 4 weeks and 65%–70% at 8 weeks on Bort + Pred treatments (Figure 1B). We did not observe significant differences in serum anti-AAV9-IgG when using Pred at high (0.75 mg/kg) or low (0.5 mg/kg) dose combined with Bort (Figure 1B) or Rap (Figure 1D). We therefore further tested the Rap + Pred approach, using Rap at different doses in combination with low-dose Pred by i.p. or oral administration (Figure 1D). Our data showed that the Rap + Pred treatments led to a similar significant reduction of anti-AAV9-IgG when using Rap at the 2 mg/kg dose or a dose 2-fold lower (1 mg/kg) or when using Pred via an i.p. or oral route (Figure 1E).

### Decrease in the Frequency of Bone Marrow B Cells and Plasma Cells following Rap + Pred Treatment

**Figure 2:**
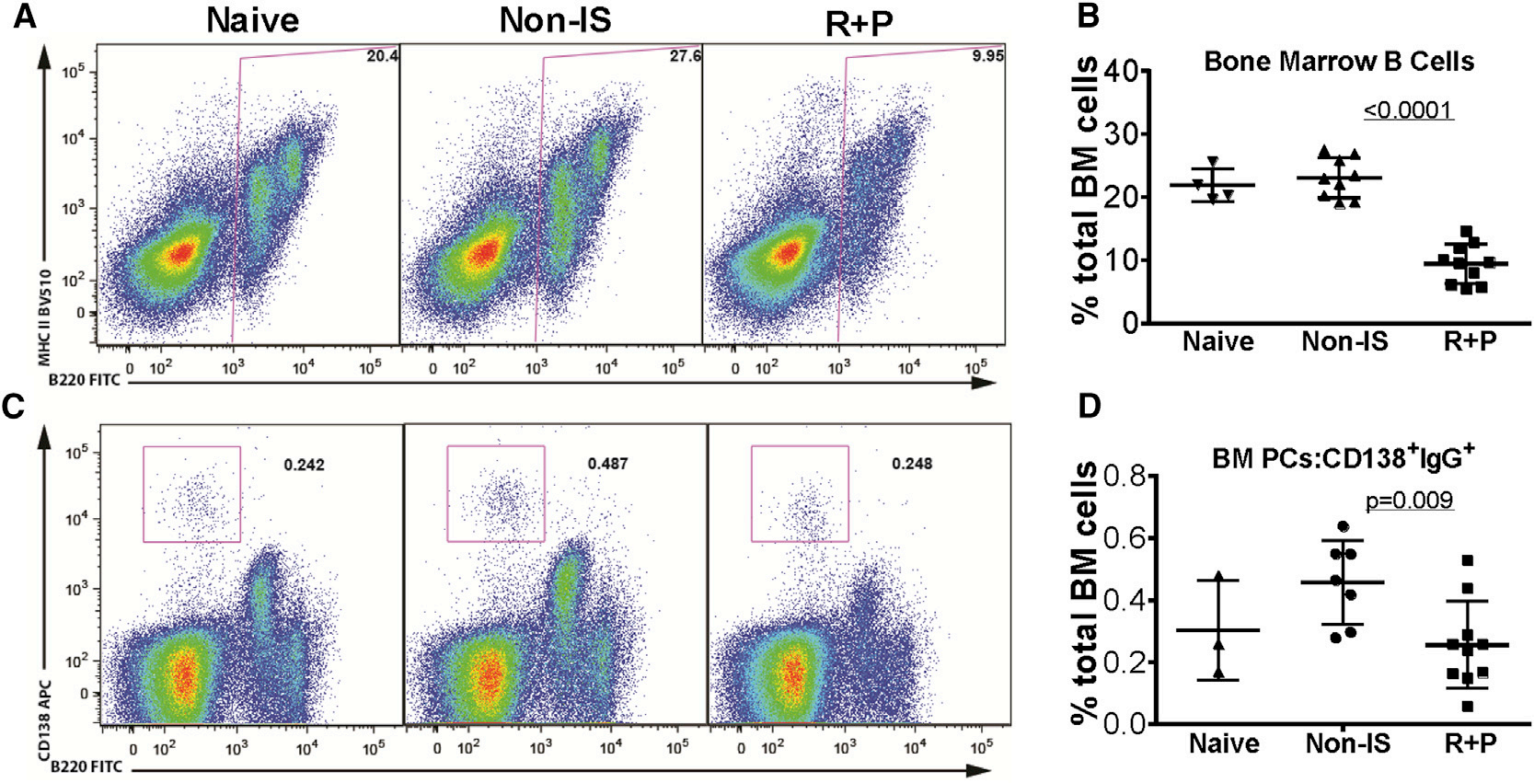
Differential Impacts of Rap + Pred on Bone Marrow B Cells and Plasma Cells (A–D) WT mice were immunized with an i.p. injection of rAAV9 vector, and then they were treated with rapamycin (R, 2 mg/kg, every other day) and prednisolone (P, 0.75 mg/kg, daily) via i.p. injection, beginning at 4 weeks post-immunization. Controls were matched naive and AAV9-immunized mice without IS treatment. At 8 weeks on IS treatment, bone marrow (BM) cells were assayed by flow cytometry for (A and B) B cells (B220^+^MHCII^+^) and (C and D) plasma cells (PC, B220^−^CD138^+^). Naive, non-immunized WT mice; non-IS, AAV9-immunized WT mice without IS treatment; R + P, AAV9-immunized mice treated with R + P.

**Figure 3:**
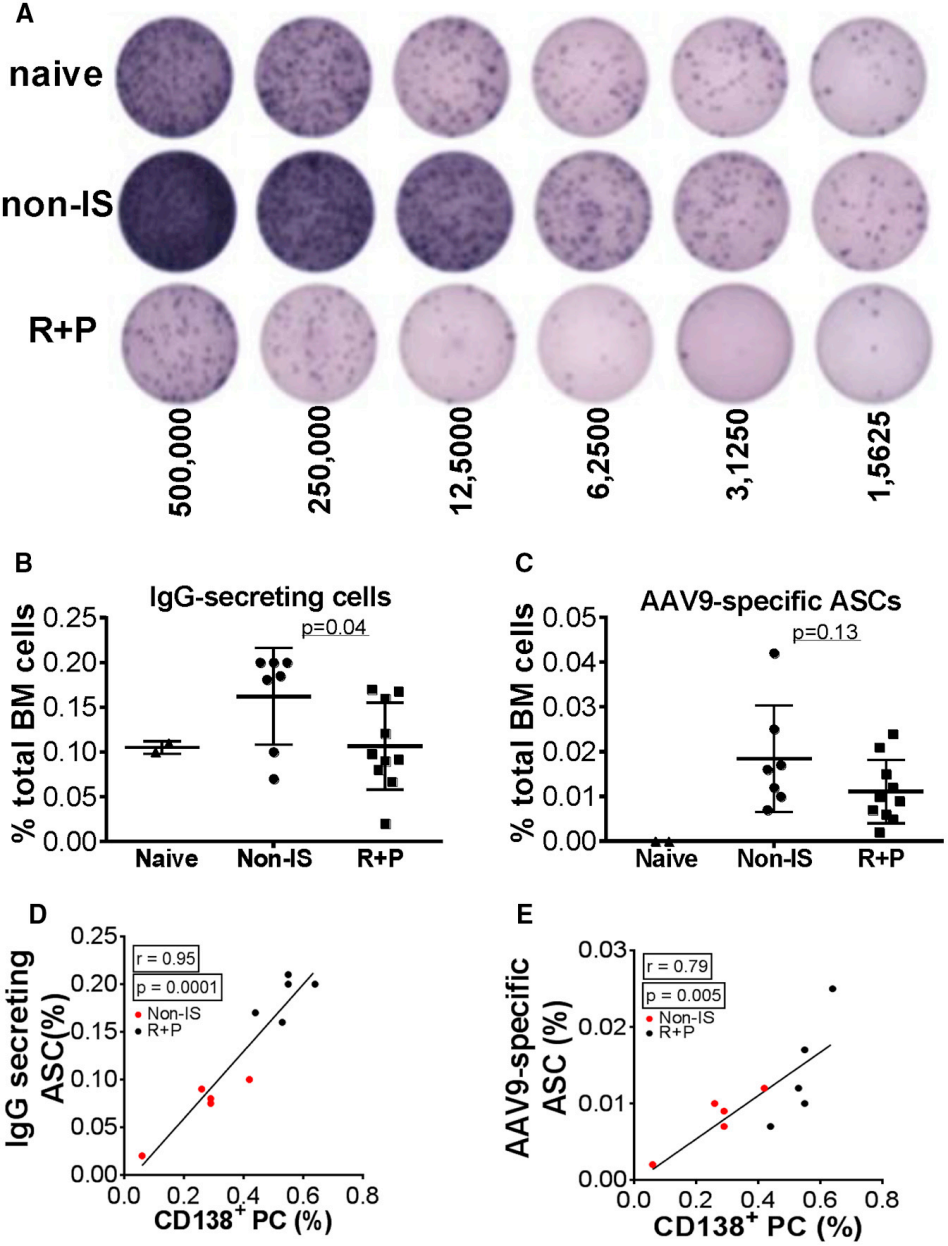
Differential Impacts of Rap + Pred on Bone Marrow Antibody-Secreting Cells (A–E) WT mice were immunized with an i.p. injection of rAAV9 vector, and then they were treated with rapamycin (R, 2 mg/kg, every other day) and prednisolone (P, 0.75 mg/kg, daily) via i.p. injection (R + P), beginning at 4 weeks post-immunization. Controls were matched naive and AAV9-immunized mice without IS treatment. Bone marrow (BM) cells were assayed at 8 weeks of IS treatment by ELISpot for (A, B, and D) IgG-secreting and (C and E) AAV9-Ab-secreting plasma cells (PCs). (B and C) p = 0.04 and p = 0.13 R + P versus non-IS. Naive, non-immunized WT mice; non-IS, non-IS-treated AAV9-immunized mice; R + P, AAV9-immunized mice treated with R + P.

To determine whether Rap + Pred treatment had inhibitory effects on antibody-producing cells in the bone marrow, flow cytometry was used to compare the percentages of bone marrow B cells (B220 and MHCII) and plasma cells (B220 and CD138) in Rap + Pred, Non-IS, and AAV naive mice (Figure 2). Interestingly, we found that Rap + Pred treatment had no effect on the total number of bone marrow cells among the three groups (Figure S1); rather, Rap + Pred treatment significantly and selectively reduced the frequency and total number of cells belonging to the B cell lineage (B220^+^MHCII^−/+^; Figures 2A and 2B) and of bone marrow-resident plasma cells (B220^−^CD138^+^; Figures 2C and 2D). Importantly, in side-by-side comparisons, functional analyses of bone marrow Ab-secreting cells using the enzyme-linked immunospot (ELISpot) method also showed reduced frequencies of IgG-secreting cells in Rap + Pred-treated mice (Figures 3A and 3B), and a trend also was evident when assessing AAV9-specific IgG-secreting cells (Figure 3C), though the difference did not reach statistical significance. In all mice studied, plasma cell frequencies as measured by phenotypic analysis were highly positively correlated to the frequencies of Ab-secreting cells measured by ELISpot, for total IgG-producing cells (Figure 3D) and for AAV9 capsid-specific IgG-producing cells (Figure 3E).

### Selective Impacts of Rap + Pred Regimen on Splenocytes: Inhibition of B Cell Activation and CD4^+^ T Cell Help

**Figure 4:**
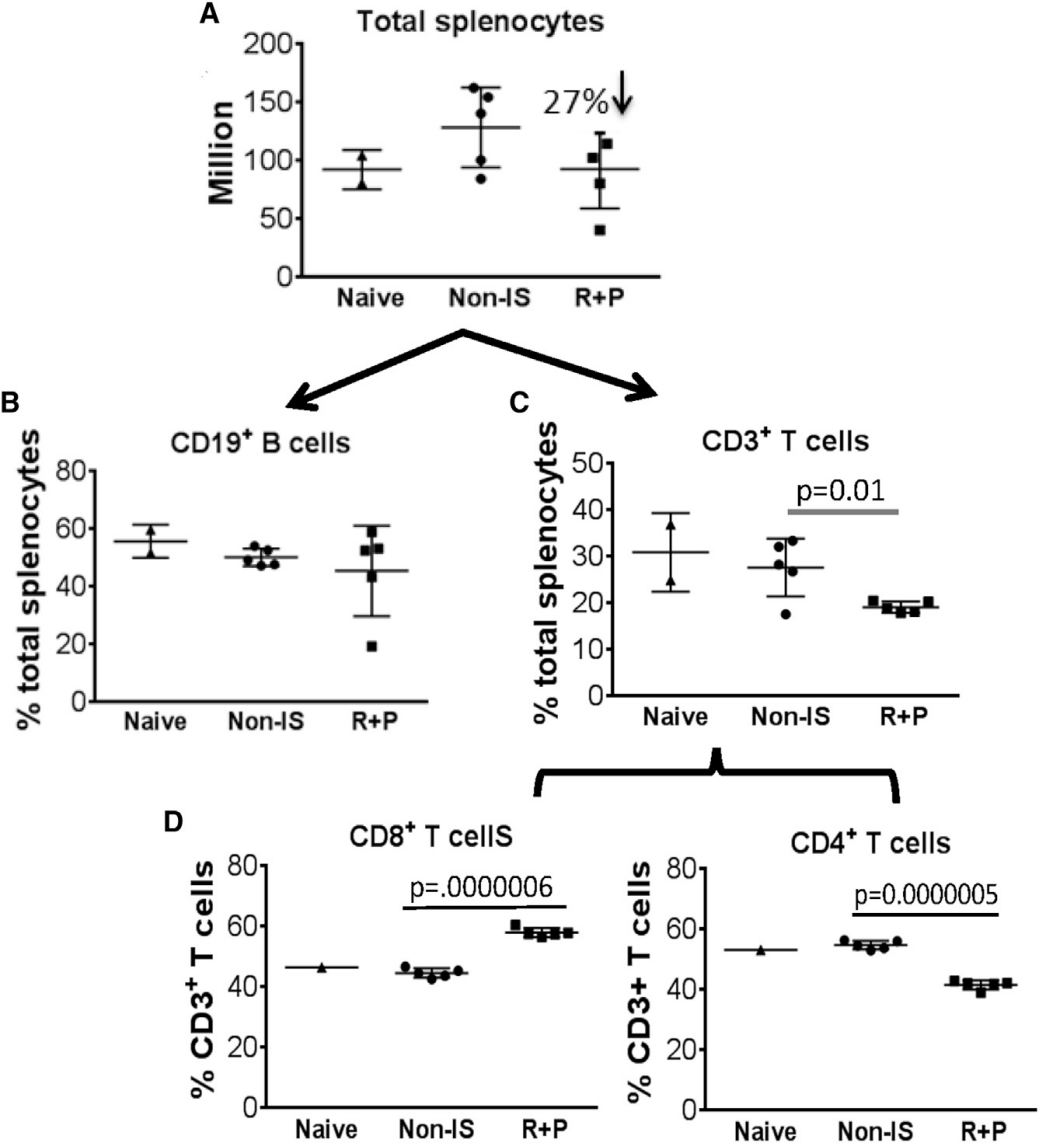
Effects of Rap + Pred on Splenocytes (A–D) WT mice were immunized with an i.p. injection of rAAV9 vector, and then they were treated with rapamycin (R, 2 mg/kg, every other day) and prednisolone (P, 0.75 mg/kg, daily) via i.p. injection, beginning at 4 weeks post-immunization. Controls were matched naive and AAV9-immunized mice without IS treatment. At 8 weeks on IS treatment, splenocytes were assayed by flow cytometry for CD3, CD4, and CD19 to determine (A) total splenocytes, (B) B cell frequencies, (C) T cell frequencies, and (D) frequencies of CD8^+^ and CD4^+^ T cell subsets. Naive, non-immunized WT mice; non-IS, AAV9-immunized WT mice without IS treatment; R + P, AAV9-immunized mice treated with R + P.

**Figure 5:**
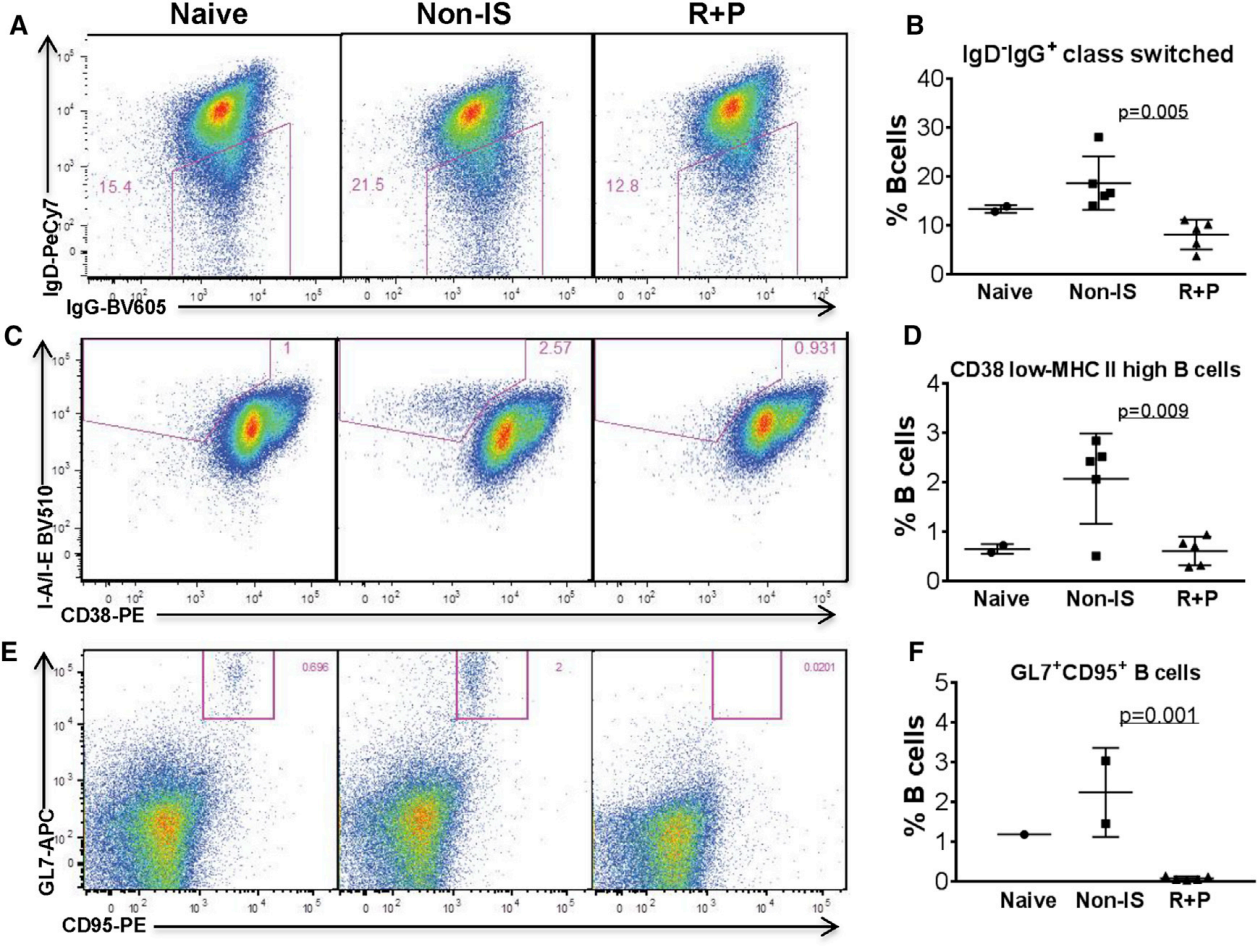
Effects of Rap + Pred on Splenic B Lymphocytes (A–F) WT mice were immunized with an i.p. injection of rAAV9 vector, and then they were treated with rapamycin (R, 2 mg/kg, every other day) and prednisolone (P, 0.75 mg/kg, daily) via i.p. injection (R + P), beginning at 4 weeks post-immunization. Controls were matched naive and AAV9-immunized mice without IS treatment. At 8 weeks on IS treatment, splenocytes were assayed by flow cytometry for CD19, IgG, IgD, CD38, GL7, and CD95. (A and B) IgD^−^IgG^+^ class switch, (C and D) CD38^low^/MHCII^high^ B cell frequencies, and (E and F) GL7^+^CD95^+^ B cell frequencies are shown. Naive, non-immunized WT mice; non-IS, AAV9-immunized WT mice without IS treatment; R + P, AAV9-immunized mice treated with R + P.

At 8 weeks on IS, flow cytometry was performed to determine the impacts of Rap + Pred on splenic lymphocyte subsets and on the priming and/or generation of antibody-producing B cells in the spleen. As expected, our data showed that the Rap + Pred combination led to a 27% decrease in total splenocytes when compared with rAAV-immunized mice that were not given IS treatment (Figure 4A); but, surprisingly, significant deficits in B cell numbers, or frequency, were not observed (Figure S1; Figure 4B). Further phenotypic analyses revealed, however, that Rap + Pred treatment was able to specifically reduce the frequencies of IgD^−^IgG^+^ class-switched B cells (Figures 5A and 5B) and B cells carrying an MHCII^high^/CD38^low^ activated (Figures 5C and 5D) or GL7^+^CD95^+^ germinal center (GC) phenotype (Figures 5E and 5F). Our data suggest that, in spleen, Rap + Pred treatment may have selective effects on B cell priming or activation for the formation of Ab-secreting cells. When directly compared, the most notable phenotypic difference for splenic B cells between Rap + Pred versus Pred alone also was related to the frequency of GL7^+^CD95^+^ GC B cells (Figures S2A and S2B).

**Figure 6:**
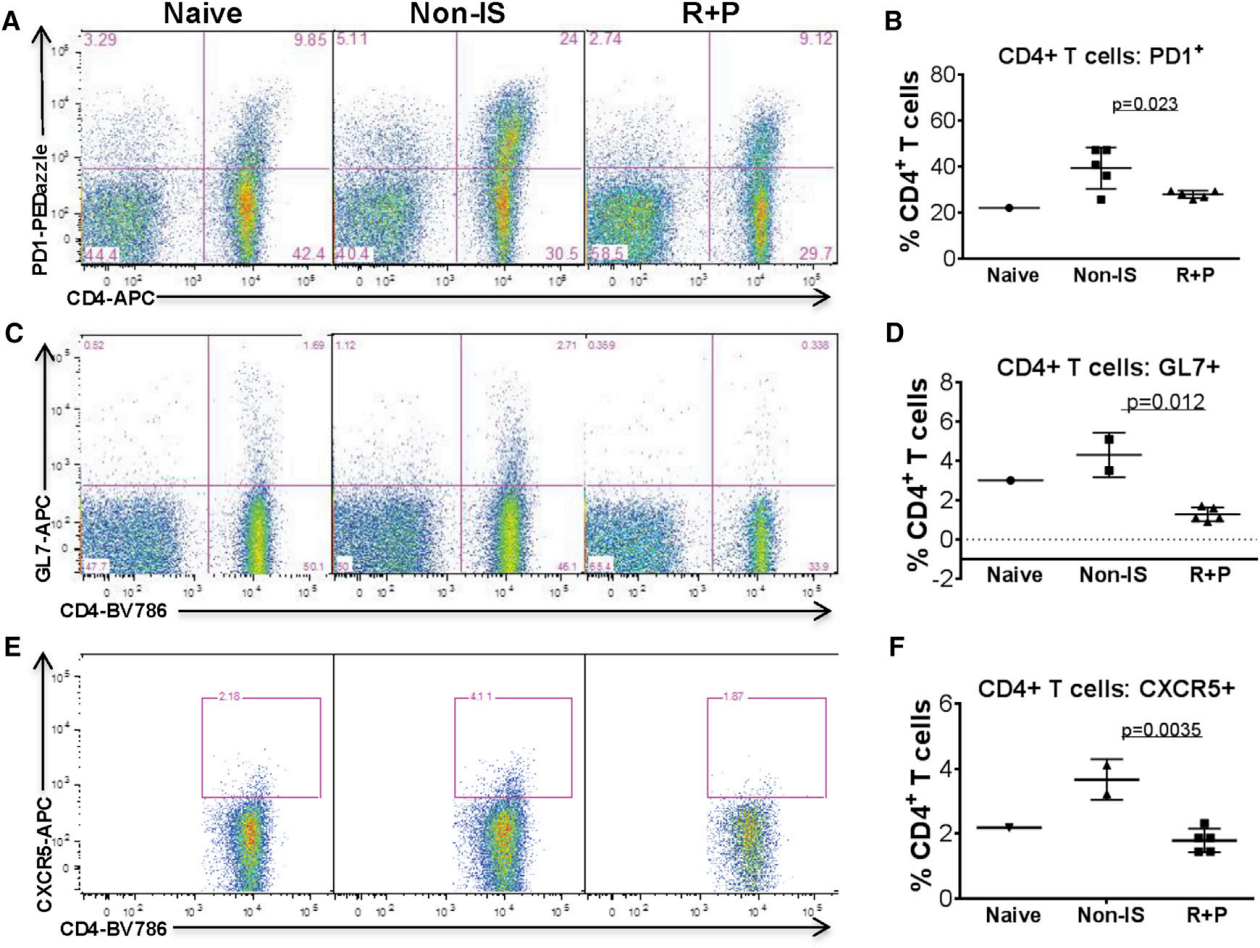
Effects of Rap + Pred on Splenic T Lymphocytes (A–F) WT mice were immunized with an i.p. injection of rAAV9 vector, and then they were treated with rapamycin (R, 2 mg/kg, every other day) and prednisolone (P, 0.75 mg/kg, daily) via i.p. injection, beginning at 4 weeks post-immunization. Controls were matched naive and AAV9-immunized mice without IS treatment. At 8 weeks on IS treatment, splenocytes were assayed by flow cytometry for CD4, PD1, GL7, and CXCR5. (A and B) CD4^+^PD1^+^ T cell frequency, (C and D) CD4^+^GL7^+^ T cell frequency, and (E and F) CD4^+^CXCR5^+^ T cell frequency are shown. Naive, non-immunized WT mice; non-IS, AAV9-immunized WT mice without IS treatment; R + P, AAV9-immunized mice treated with R + P.

It is important to note that the reduction of splenocyte numbers observed in Rap + Pred-treated mice was overall due to the decrease in CD3^+^ cells, T cells (Figures 4C and 4D). Further, the decrease in T cells was largely attributable to a selective loss of CD4^+^ T cells (Figures 4C and 4D), which led to a significant decrease in CD4:CD8 ratios. No significant changes were detected in total CD8+ cell numbers between Rap + Pred- and non-IS-treated mice. Among those remaining, there was a slight decrease in the proportion of CD4^+^ T cells expressing CD44, a marker of antigen experience, and of CD44^+^CD62L^−^ memory subsets, but these changes were not statistically significant (data not shown). Rather, the most apparent phenotypic changes observed for splenic CD4^+^ T cells in Rap + Pred mice involved the decreased expression of the co-inhibitory receptor Programmed Death-1 (PD1) (Figures 6A and 6B), activation marker GL7 (Figures 6C and 6D), and chemokine receptor CXCR5 (Figures 6E and 6F). The modulation of splenic T cell expression of GL7 and CXCR5, two markers expressed on germinal center T cells, also appeared to be the defining characteristic of Rap + Pred when compared to Pred alone in a limited number of animals (Figures S2C–S2F). When taking into account total cell numbers, we determined that, while Rap + Pred treatment resulted overall in a global reduction of splenic CD4^+^ T cells, there was an increased propensity to reduce CD4^+^ T cells expressing PD1 (Figures 6A and 6B), GL7 (Figures 6C and 6D), and CXCR5. For all the mice, the percentage of splenic CD4^+^ T cells expressing GL7 was highly correlated to the percentage expressing CXCR5 (r = 0.8), irrespective of treatment group.

## Discussion

In this study, we have identified an effective immunomodulation regimen, combining Rap and Pred, for depleting pre-existing anti-AAV9 Abs in a mouse model. We tested the three IS agents Rap, Bort, and Pred. Rap is an anti-tumor IS drug that is known to suppress T and B cell proliferation via mTOR inhibition.^32^ Bort is a proteasome inhibitor and was initially approved for the treatment of multiple myeloma, a cancer of plasma cells.^33^ Pred belongs to the family of cortical steroid drugs that inhibit broad immune components with a long proven efficacy in treating inflammation.^34^, ^35^, ^36^ Not surprisingly, using Rap, Bort, or Pred individually had no effect in serum anti-AAV9-IgG levels, while combining Rap or Bort with Pred resulted in a significant reduction in serum anti-AAV9 Abs, with optimal Ab depletion by the Rap + Pred regimen. These data support our hypothesis that effective depletion of pre-existing Abs requires broad immune system targeting.

Notably, while AAV9 Abs were the target of interest and used as the marker in this study, the Rap + Pred regimen has broad impacts on Abs in general. Therefore, it would be applicable to clinical applications of all viral vector-mediated gene therapy approaches, allowing treatment in patients who have pre-existing Abs and re-administration if needed. It is important to point out that the mouse model used in this study was a model of acute immune response to AAV9, with much higher Ab levels than the naturally occurring pre-existing AAV9 Abs typically observed in humans^9^ and in primates.^37^ It is, therefore, likely that depleting naturally occurring pre-existing AAV Abs would require significantly shorter Rap + Pred treatment when administered in humans.

Both Rap and Pred are approved drugs for humans, and the doses used in this study were determined based on those used in clinical application. The treatment regimen is, therefore, highly translatable to depleting pre-existing AAV Abs in humans. Given the nature of Rap and Pred as IS agents, it is important to minimize the potential for side effects in clinical applications. In this study, there were clearly dosage windows wherein the Rap + Pred treatments at lower doses were as effective as higher ones. The data enabled us to identify the optimal (minimal) doses of Rap + Pred for maximal effect, while presenting minimal risks in humans.

While the mechanisms remain unclear, this study demonstrates that effective Ab depletion can be achieved via a combination of broad and selective immunomodulation. The observed reduction of plasma cell frequency, decreased class-switching, and GC formation in Rap + Pred-treated mice indicate that this IS combination likely impacts multiple stages of B cell development, activation, and survival. This suggests that the effective Ab depletion attained by Rap + Pred may be a consequence of a better tuned IS regimen. Efficacy was only attainable in the circumstance where Pred, a potent generalized immunosuppressant, was paired with either the proteasome inhibitor Bort or the mTOR inhibitor Rap, forming a complementary ability to target immune populations at later stages of activation and differentiation. While cortical steroids are known broad IS agents, previous studies using corticosteroid alone have shown no detectable impact on Ab responses after immunization.^38^ Similarly, Bort has been proven efficacious for reducing anti-HLA antibodies in kidney transplant recipients, but only when used in combination therapy.^39^ These reports are consistent with our observation in this study that Pred and Bort, when used alone, had minimal effect on circulating rAAV-Ab levels.

Our data also showed that rapamycin was similarly ineffective when used alone, and, importantly, Rap + Pred had a greater inhibitory effect on circulating rAAV-Ab levels than Rap + Bort by comparison. In Rap + Pred-treated mice, the reduced circulating Ab levels also coincided with reduced class-switching, germinal center formation, and plasma cell frequencies. Such observations allude to the importance of mTOR signaling in B cell development, survival, and various stages of antigen-specific B cell activation. In bone marrow, we showed that Rap + Pred treatment had no effect on the overall cellularity of the bone marrow compartment (i.e., total bone marrow cells), but, nonetheless, it reduced the frequency of cells belonging to the B cell lineage. This observation is consistent with previous studies showing that Rap inhibits hematopoietic development and the expansion of hematopoietic progenitor cells.^40^, ^41^ Interestingly, the proportion of plasma cells targeting rAAV9 capsid (calculated as a percentage of total IgG-secreting cells) remained similar irrespective of Rap + Pred treatment. Therefore, it is unlikely that Rap + Pred would have selectively targeted rAAV-specific plasma cells. This generalized reduction of plasma cell frequency also suggests that Rap + Pred might exert its suppressive effect, in part, by modulating plasma cell survival in bone marrow, either directly or through disruption of the niche.

In contrast to the effects seen in bone marrow, this study suggests the possibility that acutely responding rAAV-specific B cells in the spleen may have increased susceptibility to Rap + Pred or were perhaps bystanders of Rap + Pred inhibition of CD4^+^ helper T cells. While the detailed mechanisms regarding the effects of Rap + Pred on rAAV-specific T cells were beyond the scope of this study, our data clearly showed increased expression of PD1, GL7, and CXCR5 in rAAV-immunized mice, and this phenotype was reversed in mice treated with Rap + Pred. PD1 is a co-inhibitory receptor that is upregulated following T cell activation, and it is increased in expression on exhausted T cells when their exposure to antigen is prolonged.^42^ Activation marker GL7 and chemokine receptor CXCR5 are each expressed by T cells participating in GC reactions within follicular compartments of the spleen.^43^ What can be highlighted using our acute Ab response model is that, in addition to the observed depletion of antibody-producing plasma B cells in bone marrow, Rap + Pred inhibition of splenic germinal center reactions had the distinction of targeting both B and T cell contributions. Recent studies have shown that mTOR signaling does, in fact, play a role in helper T cell fate decisions, leading to the generation of Th1, Th2, or Th17 responses.^42^ mTOR inhibition during priming (through treatment with rapamycin) has been shown to result in anergy or Th skewing toward a regulatory T cell fate.^42^ When taken together, our splenic data suggest that helper T cells in an activated state, such as those expressing GL7 or PD1, would be particularly susceptible to Rap + Pred and the combined targeting of pathways mediated through mTOR and complex immune pathways.^36^, ^41^ However, it is currently unclear whether helper T cells also may play a role in chronic Ab production and maintenance. Furthermore, the complex mechanisms of Ab production remain largely unclear, and more efforts are needed to reveal more potential components involved in Ab production, such as regulatory T cells, antigen-presenting cells, and major cytokine pathways.

In summary, we have identified an effective regimen for the depletion of pre-existing Abs in an artificial mouse model, combining mTOR inhibitor rapamycin and classical broad immunosuppressant prednisolone. This may have great potential for the translation of viral-mediated gene therapy approaches to clinical application in humans, allowing gene therapy treatments to be expanded to all affected patients or re-administration of vector treatment if needed. We also demonstrate that effective depletion of pre-existing AAV9 Abs requires broad immune targeting and may involve a complex mechanism via the following: (1) skewing of hematopoietic cell development, leading to alterations of immune cell compartment and the bone marrow niche; (2) direct inhibition of plasma cell survival and maintenance of Ab production in the bone marrow; and (3) reduced T cell-dependent antibody responses to rAAV capsid.

## Materials and Methods

### Animals

Animals used in this study were WT littermates of MPS IIIB knockout mice^44^ with >99.9% C57BL/6 background. The MPS IIIB mouse colony was maintained on an inbred background by backcrosses of heterozygotes in the Vivarium, the Research Institute at Nationwide Children’s Hospital. This colony has been through >30 backcrosses, alternated by a cross with WT C57BL/6 to generate heterozygotes for the next backcross. Progeny mice were genotyped by PCR. All animal care and procedures were performed strictly following the approved protocol, in accordance with the Guide for the Care and Use of Laboratory Animals. The WT mice were used in the experiments.

### Mouse Model of Pre-existing Anti-AAV9 Abs

To generate a mouse model of pre-existing Abs, 6- to 8-week-old WT mice were immunized by an i.p. injection of 1 × 10^10^ vg/kg rAAV9-CMV-hNAGLU viral vector (manufactured by SAB Tech). Serum samples were assayed by ELISA to determine total anti-AAV9 IgG levels at 4 weeks pi.

### Immunomodulation Treatments

Three approved IS drugs were used in this study, Rap, Pred, and Bort.

At 5 weeks pi, the AAV9-immunized mice were treated with Rap, Bort, and Pred, individually or in combination, at different doses (Table 1). Rap and Bort were administered every other day by i.p. injection. Pred was administered daily, either orally or via an i.p. injection. Blood samples were collected at 4 and 8 weeks on IS treatments for ELISA assays. Necropsies were performed at 8 weeks on IS, and spleen and bone marrow samples were collected for analyses. Non-IS-treated AAV9-immunized mice and naive WT mice were used as controls.

### Binding ELISA for Total Anti-AAV9-IgG

Serum samples were assayed by ELISA to determine the levels of anti-AAV9-IgG, following previously published procedures.^37^ Briefly, 96-well plates were coated with 1 × 10^10^ particles/mL empty AAV9 capsids (produced by SAB Tech) in carbonate coating buffer (antigen positive [ag^+^]) and carbonate coating buffer only (antigen negative [ag^−^]), respectively, for each sample. Following the incubation overnight at 4°C, the plates were then washed with PBS containing 0.1% Tween-20 (pH 7.4) and blocked for 1 hr with blocking buffer (5% milk in PBS containing 0.1% Tween-20). Serial dilution of serum samples in blocking buffer was added to the plates and incubated at room temperature for 1 hr. The plates were washed with PBS-T and then incubated with horseradish peroxidase-conjugated anti-mouse IgG (Sigma-Aldrich) for 1 hr at room temperature. After being washed with PBS-T, the plates were then developed with tetramethylbenzidine (TMB). The reaction was stopped by adding sulfuric acid. The absorbance was read at 450 nm on a plate reader. Serum total anti-AAV9-IgG levels are expressed as ELISA titer, based on the following calculation, such that wells with (OD_450_-ag^+^ − OD_450_-ag^−^)/OD_450_-ag^−^ ≥ 2 were considered antibody positive.

### Isolation of Splenocytes and Bone Marrow Cells

Splenocytes were isolated using mechanical disruption and suspended in RPMI media containing 10% fetal calf serum. Bone marrow samples were collected by flushing femurs and tibias with RPMI media containing 10% fetal calf serum (FCS) and filtering through a 70-μm filter.

### Flow Cytometry

Splenocytes were assayed by flow cytometry to identify immune cell subsets and their status. Overlapping panels were constructed using antibodies against mouse CD3 (fluorescein isothiocyanate [FITC], 145-2C11, BD Pharmingen), CD4 (BV786, GK1.5 BD Horizon), CD19 (PerCP/Cy5.5, 6D5, BioLegend), CD62L (BV711, MEL-14, BioLegend), CD44 (PE, IM7, BioLegend), CD27 (BV421, M-T271, BioLegend), PD1 (Pe/Dazzle594, 29F.1A12, BioLegend), CXCR5 (biotin, 2G8, BD Biosciences), GL7 (Alexa647, Gl7, BioLegend), IgD (PeCy7, 11-26c.2a, BioLegend), and IgG (BV605, poly4053, BioLegend). For CXCR5 staining, cells were first stained with a biotinylated primary CXCR5 followed by streptavidin-APC (Molecular Probes). Fluorescence minus-ones and secondary-only staining controls were used. Bone marrow cells were assayed by flow cytometry to analyze B cells, plasma cells, and their status, using antibodies against mouse CD19 (PerCP/Cy5.5, 6D5, BioLegend), B220 (FITC, RA3-6B2, BioLegend), I-A/I-E (BV510, M5/114.15.2, BioLegend), CD38 (PE, 90, BioLegend), CD138 (APC, 281-2, BioLegend), and CD27 (BV421, M-T271, BioLegend). The data were acquired using a BD-LSRII flow cytometer (BD Biosciences) and analyzed using FlowJo software (Tree Star).

### IgG-ELISpot

To assess a frequency of antibody-secreting cells (ASCs) among total bone marrow cells, IgG-ELISpot assays were performed using Mouse IgG B cell ELISpot kits (SELB004 and SEL002, R&D Systems), following the procedures provided by the manufacturer. Immunolon plates were coated with either an IgG capture antibody (to detect IgG-secreting cells) or 2 × 10^10^ vg rAAV9-CMV-hNAGLU vector (to detect AAV-specific IgG-secreting cells). Bone marrow cells were plated at 500,000, 250,000, 125,000, 62,500, 31,250, and 15,626 cells per well in RPMI containing 10% FCS and penicillin/streptomycin, and they were incubated overnight at 37°C without stimulation. The plates were washed with PBS and then incubated with biotinylated goat anti-mouse IgG polyclonal antibody at 4°C overnight. The plates were then washed before being developed using the ELISpot blue color module (SEL002, R&D Systems), and they were counted using an Immunospot Analyzer (C.T.L.). The frequencies of IgG-secreting and/or AAV-specific ASCs were calculated (number of spots/total) for each of the five dilutions, and an average was taken. The data are expressed as percentage IgG^+^ spot-forming plasma cells and percentage anti-AAV9-IgG^+^ spot-forming plasma cells.

### Data Analyses

Data were analyzed using an unpaired Student t test, Fisher’s exact test, and Pearson correlation coefficient. Significance was defined as p ≤ 0.05.

## Author Contributions

V.M.V. and A.S.M. contributed equally to this study. A.S.M. performed the in vivo experiments and antibody assays and other immunological assays. V.M.V. designed and performed the majority of the immunological analyses and co-wrote the manuscript. R.J.P. and M.C. performed the in vivo experiments and antibody assays. H.F. designed and oversaw the experiments and wrote the manuscript. D.M.M. co-designed experiments in generating the study model and edited the manuscript.

## Conflicts of Interest

H.F. and D.M.M. are co-inventors of ABO-101 and ABO-102 of Abeona Therapeutics Inc. and hold stock in the company. All other coauthors have no conflicts of interest.

## Figures and Tables

**Table 1:** Study Design

	Rap^a^ (mg/kg)	Bort^a^ (mg/kg)	Pred^b^ (mg/kg)	Number of Mice Assayed^c^		
				Pre-IS	IS-4w	IS-8w
**Experiment 1**						

non-IS	–	–	–	9	9	9
RH	2.0 (i.p.)	–	–	15	15	13
BH	–	2.0 (i.p.)	–	5	5	5
PH	–	–	0.5 (i.p.)	5	5	5
BH + PL	–	2.0 (i.p.)	0.5 (i.p.)	5	5	5
BL + PL	–	1.0 (i.p.)	0.5 (i.p.)	4	4	4
BL + PH	–	1.0 (i.p.)	0.75 (i.p.)	4	4	4
RH + PL	2.0 (i.p.)	–	0.5 (i.p.)	5	4	3
RH + PH	2.0 (i.p.)	–	0.75 (i.p.)	5	4	3

**Experiment 2**						

RH + PL	2.0 (i.p.)	–	0.5 (i.p.)	13	13	10
RL + PL	1.0 (i.p.)	–	0.5 (i.p.)	6	6	6
RL + PL(O)	2.0 (i.p.)	–	0.5 (oral)	5	5	5
non-IS	–	–	–	5	5	5

WT mice were immunized with an i.p. injection of 1 × 10^10^vg/kg rAAV9 vector and then treated with different IS agents starting 5 weeks pi. Pre-IS, prior to IS; IS-4w, 4 weeks on IS treatment; IS-8w, 8 weeks on IS treatment; non-IS, non-treated controls; R, Rap; B, Bort; P, Pred; H, high dose; L, low dose.

a Every other day.

b Daily.

c Decreases in study subjects over time were due to unexpected loss from blood draw or injections.

## Supplemental Information

Document S1. Figures S1 and S2

Document S2. Article plus Supplemental Information
