# Supplementary material for: Effective Depletion of Pre-existing Anti-AAV Antibodies Requires Broad Immune Targeting
Source: Mol Ther Methods Clin Dev. 2017 Jan 25;4:159–68. doi: 10.1016/j.omtm.2017.01.003 (PMC5363314; doi:10.1016/j.omtm.2017.01.003)
Supplement: Document S1. Figures S1 and S2 [file mmc1.pdf]

OMTM, Volume *4*

Supplemental Information

Effective Depletion of Pre-existing Anti-AAV Antibodies Requires Broad Immune Targeting

Victoria M. Velazquez, Aaron S. Meadows, Ricardo J. Pineda, Marybeth Camboni, Douglas M. McCarty, and Haiyan Fu

Supplementary Figure S1

a.

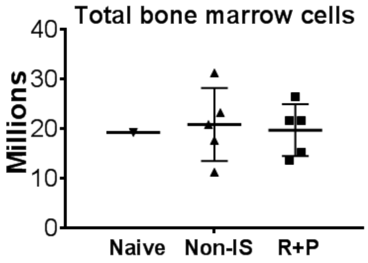

Supplementary Fig. S1. Effects of Rap+Pred on bone marrow cells. WT mice were immunized with an IP injection of rAAV9 vector, and then treated with prednisolone (0.75mg/kg, daily), or rapamycin (R, 2mg/kg, every other day) and prednisolone (P, 0.75mg/kg, daily) combined via IP injection, beginning at 4wk post immunization. Controls were matched naïve and AAV9-immunized mice without IS treatment. At 8w on IS treatment, bone marrow cells were analyzed. **Naïve:** non-immunized WT mice; **Non-IS:** AAV9-immunized WT mice without IS treatment; **R+P:** AAV9-immunized mice treated with R+P.

Supplementary Figure S2

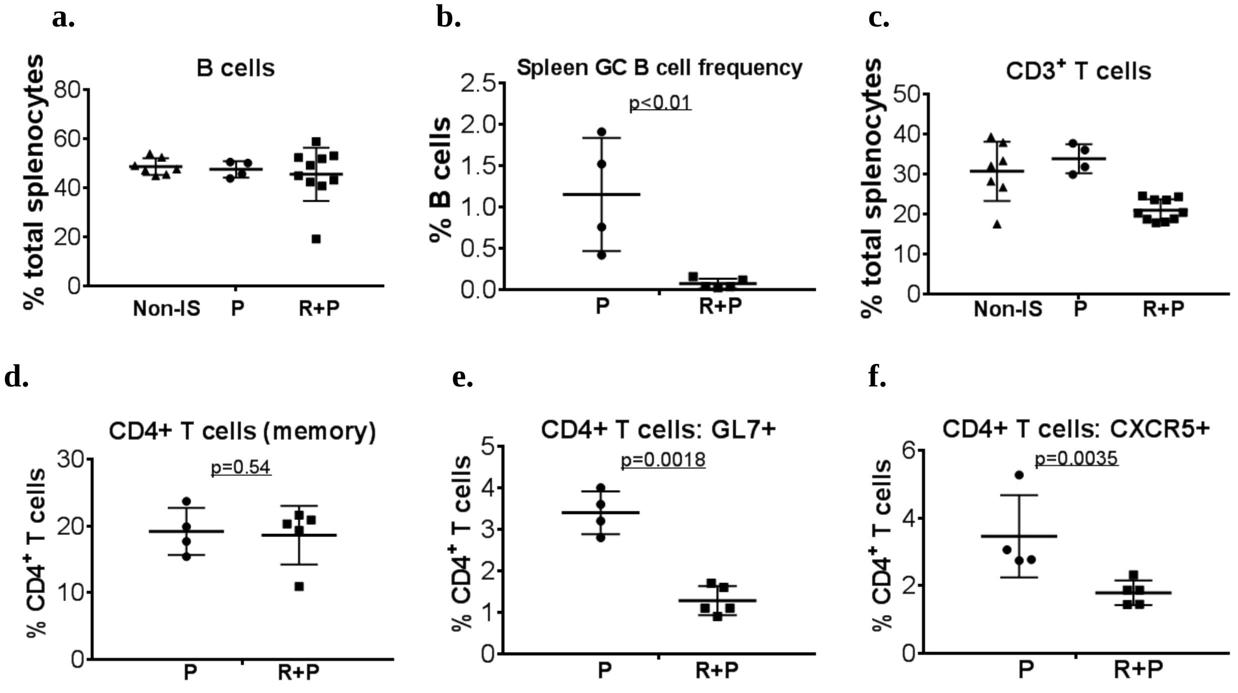

Supplementary Fig. S2. Effects of Pred alone and Rap+Pred on splenocytes. WT mice were immunized with an IP injection of rAAV9 vector, and then treated prednisolone (P, 0.75mg/kg, daily), or rapamycin (R, 2mg/kg, every other day) and prednisolone (0.75mg/kg, daily) combined via IP injection (R+P), beginning at 4wk post immunization. At 8w on IS treatment, splenocytes were assayed by flow cytometry. **a**: B cell frequencies; **b**: germinal center B cell frequency**;** **c**: T cell frequency; **d**: CD4+ memory T cell frequency; **e.** CD4+GL7+ Tcell frequency; **f.** CD4+CACR5+ T cell frequency. **Non-IS:** AAV9-immunized WT mice without IS treatment; **P:** AAV9-immunized mice treated with Prednisolone; **R+P:** AAV9-immunized mice treated with R+P.
